# Double-Pedicled Vertical Rectus Abdominis Myocutaneous Flap for Sternal Dehiscence Due to Deep Sternal Wound Infection: The Twelfth Intercostal Artery Perforator as an Additional Pedicle

**DOI:** 10.3400/avd.cr.25-00024

**Published:** 2025-07-02

**Authors:** Ryohei Ishiura, Kohei Mitsui, Kanako Danno, Kento Hosomi, Chihena Hansini Banda, Yasuhisa Urata, Mitsunaga Narushima

**Affiliations:** 1 Department of Plastic and Reconstructive Surgery, Mie University Hospital, Tsu, Mie, Japan; 2 Department of Cardiovascular Surgery, Mie Central Medical Center, Tsu, Mie, Japan

**Keywords:** VRAM, sternal dehiscence, deep sternal wound infection

## Abstract

A 50-year-old male with diabetes mellitus, who experienced recurrent sternal dehiscence secondary to a deep sternal wound infection, failed to respond to treatment with both pectoralis major muscle and greater omental flaps. Consequently, we performed a vertical rectus abdominis muscle flap in a double-pedicle fashion, utilizing the internal mammary artery and the 12th intercostal artery perforator. This intervention successfully addressed the condition. This novel technique offers an excellent therapeutic option for managing this life-threatening complication.

## Introduction

Sternal dehiscence due to deep sternal wound infection (DSWI) is one of the most serious postoperative complications of cardiac surgeries; however, its treatment is still challenging for plastic surgeons. The incidence of sternal wound complications following median sternotomy is reported to be between 0.2% and 6.1%,^1,2)^ and the associated mortality rate is reported to be between 14% and 47%.^3)^ Regarding treatment methods for sternal dehiscence due to DSWI, flap transfer is one of the most common and effective methods.^4)^ Among several flaps, the pectoralis major muscle flap (PMMF) and greater omental flap (GOF) are the workhorses for reconstruction. However, sometimes, especially in cases of immunocompromised patients with DSWI accompanied by prosthetic graft infection, it is not rare to have recurrence of DSWI after reconstruction with flaps. In cases where PMMF and GOF have already been transferred, another myocutaneous flap transfer would be required.

The vertical rectus abdominis musculocutaneous (VRAM) flap is one of the ideal alternative flaps to the PMMF and GOF. However, the VRAM flap has a major drawback in DSWI patients. Due to infection, sometimes its main pedicle, the internal mammary artery (IMA), is not reliable. Several authors have reported that the VRAM flap in DSWI patients requires supercharging, an additional arterial anastomosis to donor vessels around the defects, to transfer in a double-pedicle fashion.^5,6)^ The main candidates for supercharging recipient vessels are the contralateral IMA or the intercostal artery, but, depending on the severity of the infection, these recipient arteries are also not reliable. Moreover, there is a risk of losing candidate donor vessels for a free flap in the possible case of recurrence. To avoid these uncertain risks, we performed a reconstruction of the sternal dehiscence due to DSWI with a double-pedicled VRAM flap without supercharging, using the IMA and the 12th intercostal artery perforator (T12ICAP) as flap pedicles, and report it here.

## Case Report

**Figure 1:**
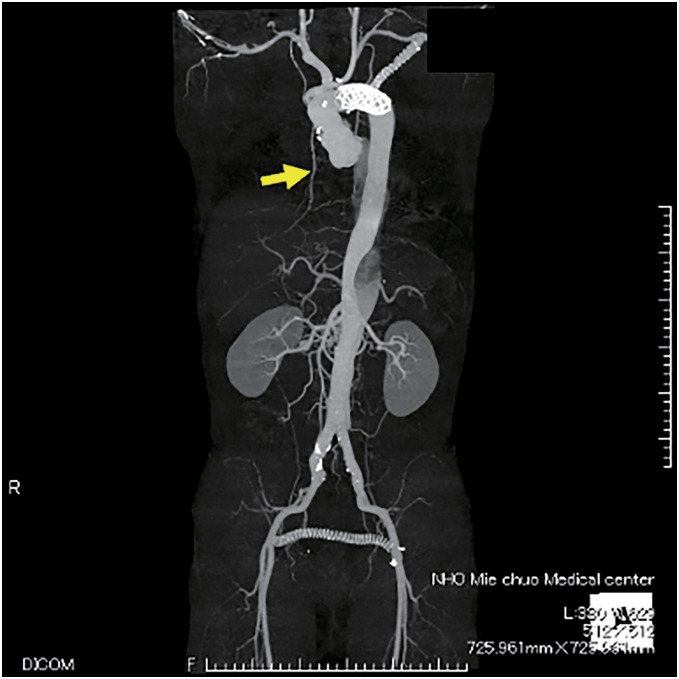
Fig. 1 Preoperative CT angiography. Yellow arrow shows the right IMA enhanced. CT: computed tomography; IMA: internal mammary artery

**Figure 2:**
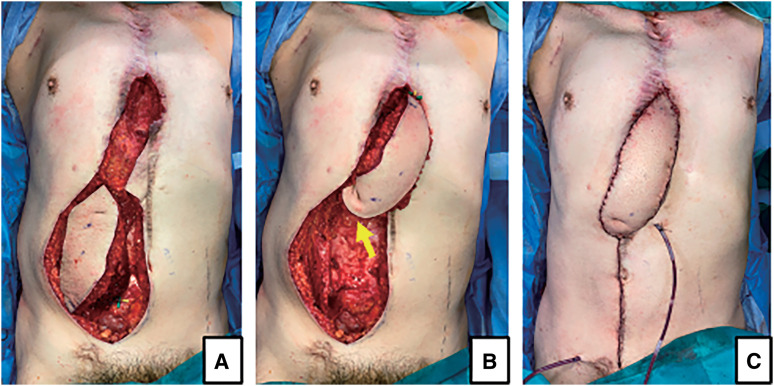
Fig. 2 (**A**) A right VRAM flap was elevated for the reconstruction of sternal dehiscence. (**B**) The yellow arrow shows the T12ICAP included in the flap. (**C**) Immediate postoperative photograph. T12ICAP: 12th intercostal artery perforator; VRAM: vertical rectus abdominis musculocutaneous

**Figure 3:**
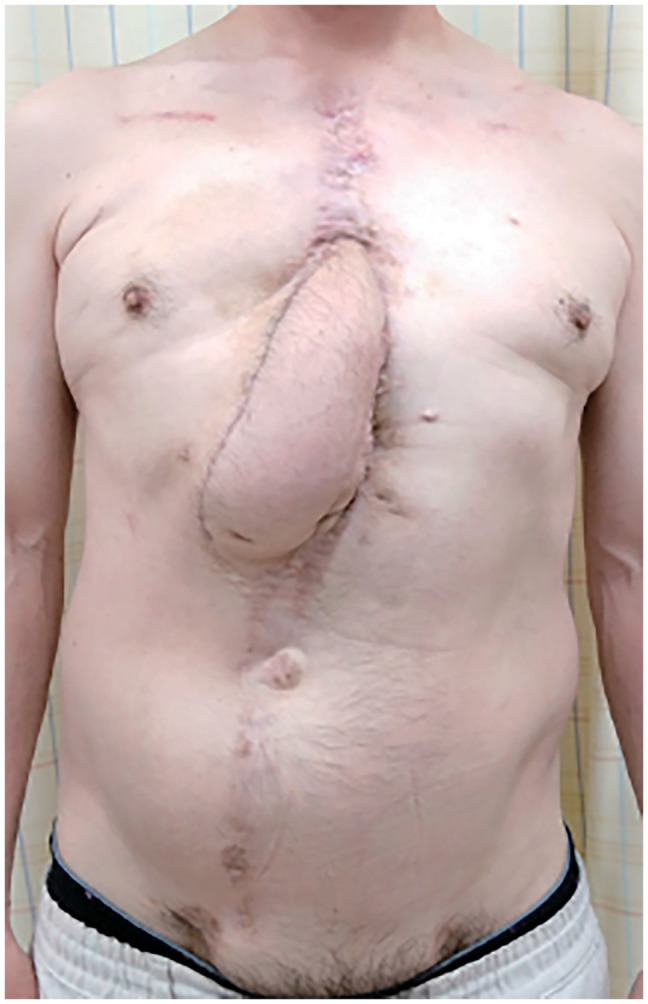
Fig. 3 Two years postoperative photograph. The flap survived completely. No recurrence was shown.

**Figure 4:**
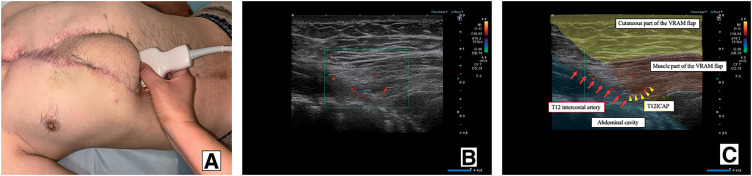
Fig. 4 (**A**) Postoperative color Doppler ultrasonography was performed to evaluate the flow of T12ICAP. (**B**, **C**) Postoperative assessment confirmed reliable perfusion through the T12ICAP to the transferred VRAM flap. T12ICAP: 12th intercostal artery perforator; VRAM: vertical rectus abdominis musculocutaneous

A 50-year-old male with diabetes mellitus experienced sternal dehiscence due to DSWI. At the age of 46, he had a Stanford type A acute aortic dissection and underwent total arch replacement, thoracic endovascular aortic repair, and femorofemoral bypass. The postoperative cause was uneventful; however, at the age of 47, he developed DSWI. Replacement of grafts was high risk due to his vessel’s condition, and debridement and omental flap transfer onto the graft were performed. The patient received meropenem intravenously for 2 weeks prior to the transfer of the GOF. Despite obtaining multiple samples before antibiotic therapy and preoperatively, no bacteria were cultured. At the age of 49, due to a 2nd recurrence of DSWI and rupture of the graft, he underwent repair of the ruptured graft and open drainage and debridement. Fortunately, his life was saved; however, his prosthetic graft was exposed, and it was difficult to control DSWI and the sternal dehiscence. After this event, 4 months of repeated negative pressure wound therapy with instillation and dwelling (NPWTi-d), open drainage, and debridement settled the DSWI, and PMMF was transferred to cover the prosthetic graft and close the sternal dehiscence. He also received daptomycin intravenously after the surgery for a week. However, a week after PMMF transfer, discharge flowed out along with the suction drain, which had been inserted during flap transfer surgery, resulting in recurrence of the sternal dehiscence. Repeated NPWTi-d and multiple debridements were performed for the following 10 months, and we planned reconstruction of the sternal dehiscence with a VRAM flap. Preoperative computed angiography (CTA) showed an enhanced IMA; however, due to the long history of DSWI, it was difficult to evaluate whether the feeder vessel flow was antegrade or retrograde (**Fig. 1**). Even if the flow was intact, it is known that without supercharging, the IMA does not provide enough vascular supply for the whole VRAM flap. The dilemma in this case was repeated by DSWI. In the case of recurrence after VRAM flap transfer with supercharging, the patient would have lost one of the candidates for recipient vessels for free flap transfer in a potential future surgery. To overcome these issues, we planned to perform a double-pedicled VRAM flap without supercharging, using the IMA and T12ICAP as flap pedicles for reconstruction of the sternal dehiscence. The double-pedicled VRAM flap was harvested from the right side. The rectus abdominis muscle was transected at the level of the arcuate line on the caudal side. On the cranial side, the muscle was not transected, and T12ICAP was detected during flap elevation and included in the flap as an additional pedicle (**Fig. 2**). The flap was rotated counterclockwise and transferred to the defect. The flap survived completely, and the postoperative course was uneventful over a 2-year follow-up period (**Fig. 3**). Using color Doppler ultrasonography (Aplio i700; Canon Medical Systems, Tochigi, Japan), postoperative assessment confirmed reliable perfusion through the T12ICAP to the transferred VRAM flap (**Fig. 4**).

## Discussion

After the 1st report of median sternotomy by Milton in 1897 and its reintroduction by Julian in 1957, the technique has become the most common access method in cardiac surgeries.^7)^ Sternal dehiscence caused by DSWI is a life-threatening complication following cardiac surgery, presenting significant challenges for treatment. The incidence of sternal wound complications after median sternotomy ranges from 0.2% to 6.1%,^1,2)^ with mortality rates varying between 14% and 47%.^3)^ One of the most common and effective treatments is flap transfer. Among various types of flaps, PMMF and GOF are considered the most popular for reconstructing the area. However, in certain cases, particularly in patients with immunosuppression and prosthetic graft infections, there is a risk of recurrence of DSWI even after flap reconstruction. When PMMF and GOF have already been used, a 3rd myocutaneous flap transfer may be required for successful wound closure.

The VRAM flap has abundant muscle volume for covering exposed prosthetic grafts, and its location makes it easy to access the sternal dehiscence. The flap is one of the ideal flaps for chest wall reconstruction; however, under DSWI conditions, surgeons are reluctant to adopt it. The main reason is that the pedicle of the VRAM flap, the IMA, is vulnerable to DSWI. It is likely to fail in flap transfer with a damaged vascular pedicle; however, the problem is the difficulty in evaluating its flow with CTA. Since the deep inferior epigastric artery (DIEA) is connected to the IMA, CTA cannot describe whether the flow in the IMA is antegrade from the IMA or retrograde from the DIEA. Some authors have reported partial necrosis of the VRAM flap in sternal dehiscence reconstruction cases,^8)^ and supercharging to the contralateral IMA or the transverse cervical artery has been reported.^5,6)^ Wimmers and Lifchez reported the intercostal artery-based rectus abdominis muscle flap, and in the article, they used T12ICAP as a pedicle.^9)^ In our case, we harvested the VRAM flap in a double-pedicle fashion with IMA and T12ICAP. With this technique, T12ICAP reinforces the flap vascularity instead of supercharging. In complicated cases, the recurrence rate of DSWI is relatively higher than in simple DSWI cases, and it is important to preserve candidates for recipient vessels for another flap with supercharging or free flaps. This technique provides a reliable VRAM flap in a simple way without sacrificing recipient vessels. However, this procedure has certain limitations. The main limitation is that due to the difficulty in evaluating the flow of the IMA, it is difficult to assess whether the flap requires T12ICAP as an additional pedicle. However, even if the IMA is intact, flap harvest including T12ICAP does not take additional time and effort.

We believe this case report could provide valuable insights for future research involving larger patient groups and extended follow-up periods to confirm the benefits and fine-tune the criteria for this reconstructive method. Additionally, it could serve as a useful resource for reconstructive surgeons, helping them expand their surgical techniques to manage similar cases and enhance patient outcomes.

## Conclusion

We report a case of VRAM flap using IMA and T12ICAP as its pedicles for the reconstruction of sternal dehiscence due to DSWI. VRAM flap tends to be avoided as a reconstructive option in view of the pedicle reliability for the dehiscence. With our technique, VRAM flap would be a safe candidate for the reconstruction of sternal dehiscence.

## Declarations

### Acknowledgment

None.

### Informed consent

Consent was obtained from the patient for the publication of this report, including the use of images.

### Disclosure statement

All authors have no conflict of interest.

### Author contributions

Study conception: RI

Data collection: RI, KM, KD, KH, YU, and CHB

Analysis: RI

Investigation: RI

Manuscript preparation: RI

Critical review and revision: all authors

Final approval of the article: all authors

Accountability for all aspects of the work: all authors.
